# A Risk Prediction Score for Invasive Mold Disease in Patients with Hematological Malignancies

**DOI:** 10.1371/journal.pone.0075531

**Published:** 2013-09-26

**Authors:** Marta Stanzani, Russell E. Lewis, Mauro Fiacchini, Paolo Ricci, Fabio Tumietto, Pierluigi Viale, Simone Ambretti, Michele Baccarani, Michele Cavo, Nicola Vianelli

**Affiliations:** 1 Institute of Hematology, Department of Hematology and Clinical Oncology, “Lorenzo e Ariosto Seràgnoli” S’Orsola-Malpighi Hospital, University of Bologna, Bologna, Italy; 2 Clinic of Infectious Diseases, Department of Internal Medicine, Geriatrics and Nephrologic Diseases, S’Orsola-Malpighi Hospital, University of Bologna, Bologna, Italy; 3 Operative Unit of Microbiology, Department of Hematology, Oncology and Laboratory Medicine, S’Orsola-Malpighi Hospital, University of Bologna, Bologna, Italy; University of Wisconsin Medical School, United States of America

## Abstract

**Background:**

A risk score for invasive mold disease (IMD) in patients with hematological malignancies could facilitate patient screening and improve the targeted use of antifungal prophylaxis.

**Methods:**

We retrospectively analyzed 1,709 hospital admissions of 840 patients with hematological malignancies (2005-2008) to collect data on 17 epidemiological and treatment-related risk factors for IMD. Multivariate regression was used to develop a weighted risk score based on independent risk factors associated with proven or probable IMD, which was prospectively validated during 1,746 hospital admissions of 855 patients from 2009-2012.

**Results:**

Of the 17 candidate variables analyzed, 11 correlated with IMD by univariate analysis, but only 4 risk factors (neutropenia, lymphocytopenia or lymphocyte dysfunction in allogeneic hematopoietic stem cell transplant recipients, malignancy status, and prior IMD) were retained in the final multivariate model, resulting in a weighted risk score 0-13. A risk score of < 6 discriminated patients with low (< 1%) versus higher incidence rates (> 5%) of IMD, with a negative predictive value (NPV) of 0.99, (95% CI 0.98-0.99). During 2009-2012, patients with a calculated risk score at admission of < 6 had significantly lower 90-day incidence rates of IMD compared to patients with scores > 6 (0.9% vs. 10.6%, *P* <0.001).

**Conclusion:**

An objective, weighted risk score for IMD can accurately discriminate patients with hematological malignancies at low risk for developing mold disease, and could possibly facilitate “screening-out” of low risk patients less likely to benefit from intensive diagnostic monitoring or mold-directed antifungal prophylaxis.

## Introduction

Invasive mold diseases (IMDs) such as aspergillosis, and less commonly mucormycosis and fusariosis are a serious complication of myelosuppressive chemotherapy administered for hematological malignancies [1-3]. Patients undergoing allogeneic hematopoietic stem cell transplantation (HSCT) or remission-induction chemotherapy for acute myelogenous leukemia / myelodysplastic syndrome (AML/MDS) are at especially high risk, with 20-fold higher rates of aspergillosis compared to patients with underlying lymphoma or multiple myeloma [4]. Although diagnostic advances and new antifungal therapies have improved survival rates in patients with invasive aspergillosis [1], nearly one-third of patients still die with the infection, or have interruption of life-saving chemotherapy while the mold infection is being treated [1]. As a result, many hematologists routinely screen patients for incipient mold infection with the serum galactomannan test and high resolution computer tomography if the patient has fever, or administer mold-active antifungal prophylaxis for prolonged periods even though only a small proportion of patients (4-12%) may go on to develop a mold infection [5,6].

Risk stratification for IMD is a logical first step for identifying which patients would most likely benefit from more intensive monitoring or antifungal prophylaxis [7,8]. However, the development of an IMD risk prediction model in patients with hematological malignancies is complicated by the low overall disease prevalence, infrequently analyzed genetic risk factors related to host innate immunity, and dynamic clinical and environmental variables during their course of treatment [8,9]. Nevertheless, we hypothesized that an objective risk score for hematology patients based on easily documented demographic and clinical risk factors could have clinical utility if it accurately discriminates populations at low versus higher risk for developing IMD.

As a first step towards this goal, we retrospectively analyzed 17 candidate epidemiological and clinical risk factors for IMD in 840 patients during 2005-2008 to develop an objective risk score for proven or probable IMD. We then prospectively evaluated the performance of this risk score in 855 patients from 2009-2012. We found that a weighted risk score for IMD accurately discriminated a cohort of hematology patients at low (

< 1% incidence) versus higher (> 5% incidence) risk for mold infection, irrespective of the underlying malignancy, transplant status, and use of mold-active antifungal prophylaxis.

## Design and Methods

### Ethics statement

The study was conducted in accordance with the Declaration of Helsinki, following review by the S’Orsola-Malpighi-University of Bologna ethics committee (http://www.aosp.bo.it/content/comitato-etico). Full review was waived because of the non-interventional, observational nature of the study. As a standard protocol in our institute, all patients included in the study provided an informed consent the first day of hospitalization.

### Study Population

This study was performed at a single regional hematology center in Italy (Institute of Hematology and Clinical Oncology “Lorenzo e Ariosto Seràgnoli”, University of Bologna) during two periods. In the first study period (March 2005-December 2008), consecutive hospital admissions of patients with hematological malignancies were retrospectively analyzed for infections and IMD risk factors to develop a multivariate risk model for IMD. During the second study period (January 2009-December 2012), the performance of the risk score was prospectively analyzed in patients with a risk score calculated at the time of hospital admission, which was not reported to the treating hematologist. For each patient hospitalization, only the first infection episode was included in the analysis. Patients with hospitalizations shorter than 6 days were excluded from the analysis.

We collected data on 17 candidate predictors for IMD, which had been previously reported in the literature as risk factors for IMD in patients with hematological malignancies (Table 1). Additional data pertinent to each hospitalization and infection episode were collected from clinical records and registered on a standardized data collection form by the treating hematologist, while demographic data were extracted from an institutional centralized database. The accuracy of collected data was confirmed by a quality control procedure during data entry and with periodic reviews by 4 physicians (2 hematologists, 1 infectious diseases specialist, and 1 radiologist).

**Table 1:** Screened Risk Factors for Invasive Mold Disease.

**Variable**	**Risk Factor**	**Definitions, comments**	**References**
1	Age > 40	Related to hematologic malignancy treatment response	[20,21]
2	Profession with likely repeated exposure to fungal spores	Patient works as a farmer, mason, carpenter/construction or has outdoor work with likely spore exposures	[8]
3	Smoking habits	Current user of tobacco or marijuana	[22]
4	Prior clinical history of proven or probable mold disease	Documented within 1 year of hospital admission	[23-26]
5	History of diabetes	Diagnosis of insulin-dependent or non-insulin-dependent diabetes mellitus	[27]
6	High-dose corticosteroid treatment	0.5 mg/kg daily within 30 days prior to hospital admission	[25,28-32]
7	High-risk underlying malignancy	Diagnosis of acute myeloid leukemia/ myelodysplastic syndrome, or aplastic anemia	[4,33]
8	Malignancy status at time of admission	Underlying malignancy is not in partial or complete remission.	[4,21,34]
9	Hospital admission for high-risk chemotherapy	Patient currently receiving or admitted for chemotherapy to treat acute myeloid leukemia/ myelodysplastic syndrome, severe aplastic anemia, or for allogeneic HSCT conditioning chemotherapy	[4,33,35,36]
10	Prolonged neutropenia	Absolute neutrophil count < 500 cells/µL for greater than 10 days within 30 days prior to admission or following chemotherapy	[7,33,37,38]
11	Lymphocytopenia or probable impaired lymphocyte function at time of admission	Lymphocytopenia (or probable impaired lymphocyte function) defined as an CD4^+^ count < 50 cells/µL; or any allogeneic HSCT patient receiving cyclosporine, tacrolimus, or anti-thymocyte globulin	[25,39,40]
12	Severe acute graft versus host disease after transplantation	“Severe” graded according to Glucksberg [41] criteria	[42]
13	Severe chronic graft versus host disease at admission	“Severe” graded according to Shulman [43] criteria	[25,39]
14	Severe mucositis during hospitalization	WHO classification of Grade 3 or 4	[44]
15	Cytomegalovirus infection	Patient has evidence of active CMV infection diagnosed by pp65 antigen or quantitative PCR	[40,45]
16	Admission to a hospital room without high-efficiency particulate air (HEPA) filtration	Room does not contain central HEPA air filtration	[46,47]
17	Admission to hospital room in proximity of construction	Patient was admitted to hospital room in a ward or building with ongoing construction	[46,48,49]

### Study endpoint

The primary endpoint used for score development was documentation of proven or probable IMD within 90 days of hospitalization. Possible, probable or proven invasive aspergillosis (IA) and invasive mold disease (IMD) was defined according to the revised Mycoses Study Group and European Organization and Treatment of Cancer consensus criteria [10]. Serum galactomannan testing was routinely available at our institute after January 2007. Before this period, typical radiographic criteria as described by Cornely et al., were used to classify patients with proven or probable invasive aspergillosis [11]. In the case of non-*Aspergillus* molds not detected by galactomannan screening, diagnosis was always confirmed by histology or culture. Fluconazole (400 mg daily) was routinely administered to all patients undergoing allogeneic HSCT. Decisions regarding anti-mold antifungal prophylaxis in either non-transplant or transplant patients were at the discretion of the physicians caring for the patient.

### Statistical Analysis

Demographic data were collected as either continuous data and compared by two-tailed unpaired *t*-test or Mann-Whitney test, or as categorical variables and compared by Chi-square test for patients with or without a probable or proven IMD. Variables with more than 5% missing data were excluded from analysis. Significant variables (*P*<0.05) were entered stepwise in a multivariate logistic regression model to evaluate the relationship between each variable and IMD risk using the Wald’s statistic. Variables that maintained statistical significance by multivariate regression were then assigned a point value corresponding to the β-coefficient of that variable divided by the lowest β-coefficient of variables remaining in the regression model, and the resulting quotient was multiplied by two and rounded to the nearest whole number. Summation of the points resulted in a weighted risk score that was assigned to each patient episode retrospectively (2005-2008), or prospectively (2009-2012) at the time of hospital admission.

The relationship of the calculated risk score and IMD risk was subsequently analyzed by receiver operator curves (ROC) to define an optimal cut-off score that discriminated low, versus high-risk patients. Our provisional cut-off was a risk score associated with 5% incidence of IMD, which has been proposed as the lower incidence limit of *Aspergillus* infection justifiable for antifungal prophylaxis in hematology patients [12]. All statistical analysis was performed using SPSS version 20 (IBM, Armonk, NY) and MedCalc 12.5 (Ostend, Belgium).

## Results

### Study populations

During the retrospective study period (2005-2008), we analyzed 1,709 hospital admissions from 840 patients with hematological malignancies. Each patient contributed a median of 2 separate hospitalizations to the database (range 1-12). The most common underlying malignancies were AML/MDS (31%), lymphoma (29%), and multiple myeloma/amyloidosis (25%) of which 63% were in partial or complete remission. Nearly 40% of the hospitalizations were for chemotherapy alone (i.e. no evidence of fever or infection on admission) with 46% of these admissions proceeding to HSCT (34% autologous, 12% allogeneic). Characteristics of the 1,709 cases are summarized in Table 2.

**Table 2:** Patient Demographic Characteristics.

**Characteristic**	**2005-2008 Cohort;n=1,709 episodes(%**)	**2009-2012 Cohort;n=1,746 episodes(%**)	***P* value^a^**
Median age (range)	52 (15-84)	52 (15-87)	0.92
Sex, male	1,013 (59)	1,047 (60)	0.95
Median no. of hospitalizations (range)	2 (1-12)	1 (1-10)	0.52
**Underlying malignancy**			
Acute myeloid leukemia/myelodysplastic syndrome	527 (31)	541 (31)	0.95
Acute lymphoblastic leukemia	176 (10)	193 (11)	0.51
Chronic myelogenous leukemia	50 (3)	6 (0.3)	< 0.001
Chronic lymphocytic leukemia	19 (1)	65 (4)	< 0.001
Lymphoma	490 (29)	568 (36)	0.02
Multiple myeloma/ amyloidosis	418 (24)	332 (14)	0.001
Aplastic anemia	13 (0.8)	18 (19)	0.51
Non-neoplastic hematological disease	16 (0.9)	23 (1)	0.37
**Disease status**			
Newly diagnosed	197 (12)	192 (11)	0.67
Complete/ partial response	1030 (60)	1021 (58)	0.29
Progression/ resistance/ relapse	482 (28)	533 (31)	0.14
**Type of treatment (%)**			
Induction chemotherapy	151 (9)	335 (19)	< 0.001
Other chemotherapy ^b^	229 (13)	415 (24)	< 0.001
Rescue chemotherapy ^c^	278 (16)	204 (12)	0.43
Allogeneic HSCT	203 (12)	227 (13)	0.34
Autologous HSCT	584 (34)	334 (20)	< 0.001
No chemotherapy ^d^	264 (15)	206 (12)	0.002
Anti-mold prophylaxis^e^(systemically-active agent)	188 (11)	354 (20)	< 0.001
Empiric mold-active antifungal within 60 days of hospitalization	239 (14)	148 (8)	<0.0001

a Pearson Chi-square for nominal data, Mann-Whitney or 2-tailed Students t-test for continuous data

b Includes maintenance chemotherapy, consolidation chemotherapy

c Chemotherapy administered for relapsed disease

d Includes all admissions where chemotherapy was not administered (diagnostic, stem-cell mobilization, medical complications, etc.)

e Prescribed agents: 2005-2008: itraconazole 10%, voriconazole 0.4%, lipid amphotericin B 0.6%; Prescribed agents 2009-2012: posaconazole 11.4%, itraconazole 8%, voriconazole 0.6%, lipid amphotericin B 0.3%

During the prospective score validation study period (2009-2012), we analyzed 1,746 hospital admissions in 855 hematology patients. Each patient contributed a median of 1 hospitalization episode (range 1-10) to the database. The breakdown of underlying malignancies in the prospectively studied cohort was similar to the retrospective cohort. However, significantly fewer patients in 2009-2012 were admitted with chronic myelogenous leukemia (0.3% vs. 3%, *P*<0.001) or multiple myeloma/amyloidosis (14% vs. 24% *P*=0.001). Admissions associated with chronic lymphocytic leukemia (4% vs. 1%, *P*<0.001) and lymphoma (36% vs. 29% *P*=0.02) were slightly higher during 2009-2012. Additionally, a higher proportion of hospital admissions in prospectively studied patients were for induction (19% vs. 9%, *P*<0.001) or maintenance/ salvage chemotherapy (24% vs. 13%, *P*<0.001); reflecting the activation of new protocols in our institute during 2009-2012. Fewer patients in 2009-2012 received an autologous HSCT (20% vs. 34%, *P*<0.001), although rates of allogeneic HSCT were similar between the two study periods (13% vs. 12%, *P*=0.34).

Anti-mold antifungal prophylaxis was used more frequently in 2009-2012 (20% vs. 11%, *P*<0.001), which was largely attributed to the introduction use of posaconazole after 2009 (Table 2). The increased use of anti-mold prophylaxis was associated with a corresponding decrease in empirical antifungal therapy for molds (8% vs. 14%, *P*<0.001). The most common anti-mold antifungal prophylaxis used during 2005-2008 was itraconazole capsules or solution (10%), which was largely replaced by posaconazole during 2009-2012 (11.4%) with some continued itraconazole use (8%). Voriconazole, lipid amphotericin B formulations, or aerosolized amphotericin B formulations were infrequently administered as prophylaxis during either study period (all less than 1%).

### Risk Factors Associated with Proven or Probable IMD

Among the 17 candidate variables evaluated in the retrospective cohort, 11 were associated with IMD by univariate analysis (Table 3). These included patient occupational risk factors, the status of the underlying hematologic malignancy, variables related to the severity of underlying immunosuppression, a prior history of IMD, as well as the admission to a non-HEPA air-filtered room. However, in multivariate regression, only 4 of the 11 variables were independently associated with IMD risk: 1) Prolonged neutropenia, 2) lymphocytopenia or functional lymphocytopenia in allogeneic HSCT patients; 3) prior history of IMD, and 4) underlying malignancy that was not in partial or complete remission (Table 4).

**Table 3:** Univariate analysis of risk factors for invasive mold disease.

	**2005-2008 Cohortn=1,709 episodes**			**2009-2012 Cohortn=1,746 episodes**		
**Risk factor^a^**	**No IMD (%)n=1,650**	**IMD (%)n=59**	***P* value^b^**	**No IMD (%)n=1,691**	**IMD (%)n=55**	***P* value^b^**
1-Age >40	1264 (77)	44 (75)	0.73	1,269 (75)	37 (67)	0.13
2-At-risk profession	168 (10)	10 (17)	0.05	137 (8)	6 (11)	0.29
3-Smoker	542 (33)	20 (34)	0.65	419 (25)	13 (24)	0.50
4-Prior IMD	31 (2)	7 (12)	<0.001	42 (2)	11 (20)	< 0.001
5-Diabetic	156 (9)	10 (17)	0.03	105 (6)	3 (5)	0.55
6-Corticosteroids	312 (19)	16 (27)	0.06	192 (11)	12 (22)	0.02
7-High-risk malignancy	555 (34)	37 (63)	<0.001	552 (32)	29 (53)	0.006
8-Uncontrolled malignancy	755 (46)	47 (80)	<0.001	693 (41)	32 (58)	0.008
9-High-risk chemotherapy	512 (31)	37 (63)	<0.001	420 (25)	36 (65)	<0.001
10-Neutropenia > 10 days	596 (36)	48 (81)	<0.001	593 (35)	47 (85)	<0.001
11-Lymphocytopenia or dysfunction	415 (25)	31 (53)	<0.001	222 (13)	35 (64)	<0.001
12-Acute GVHD, grade II-IV	47 (3)	5 (8)	0.02	35 (2)	5 (9)	<0.001
13-Chronic GVHD, extensive	28 (2)	1 (2)	0.07	9 (0.5)	1 (2)	0.36
14-Mucositis, Grade III-IV	206 (12)	14 (24)	0.004	117 (7)	11 (20)	0.002
15-CMV Infection	62 (4)	4 (7)	0.18	48 (3)	5 (9)	0.02
16-Admission to non HEPA room	587 (36)	29 (49)	0.01	482 (29)	21 (38)	0.45
17-Proximity to construction	202 (12)	8 (14)	0.64	412 (24)	10 (18)	0.19

a See Table 1 for definitions

b Chi square test

**Table 4:** Multivariate regression model developed from the retrospective cohort of 1,709 hospitalizations (2005-2008).

**Variable**	**Frequency in patients with IMD (%)**	**β-coeff**	**Wald χ^2^**	***P* value**	**Hazard Ratio(95% CI)**	**Points**
Duration of neutropenia	596 (41)	1.72	21.99	< 0.001	5.60 (2.72-11.50)	4
Previous IMD	31 (9)	1.71	12.42	< 0.001	5.55 (2.14-14.41)	4
Malignancy status	755 (50)	1.53	19.46	< 0.001	4.64 (2.34-9.19)	3
Lymphocytopenia or lymphocyte dysfunction	415 (31)	0.90	9.57	0.002	2.45 (1.39-4.34)	2

**Figure 1:**
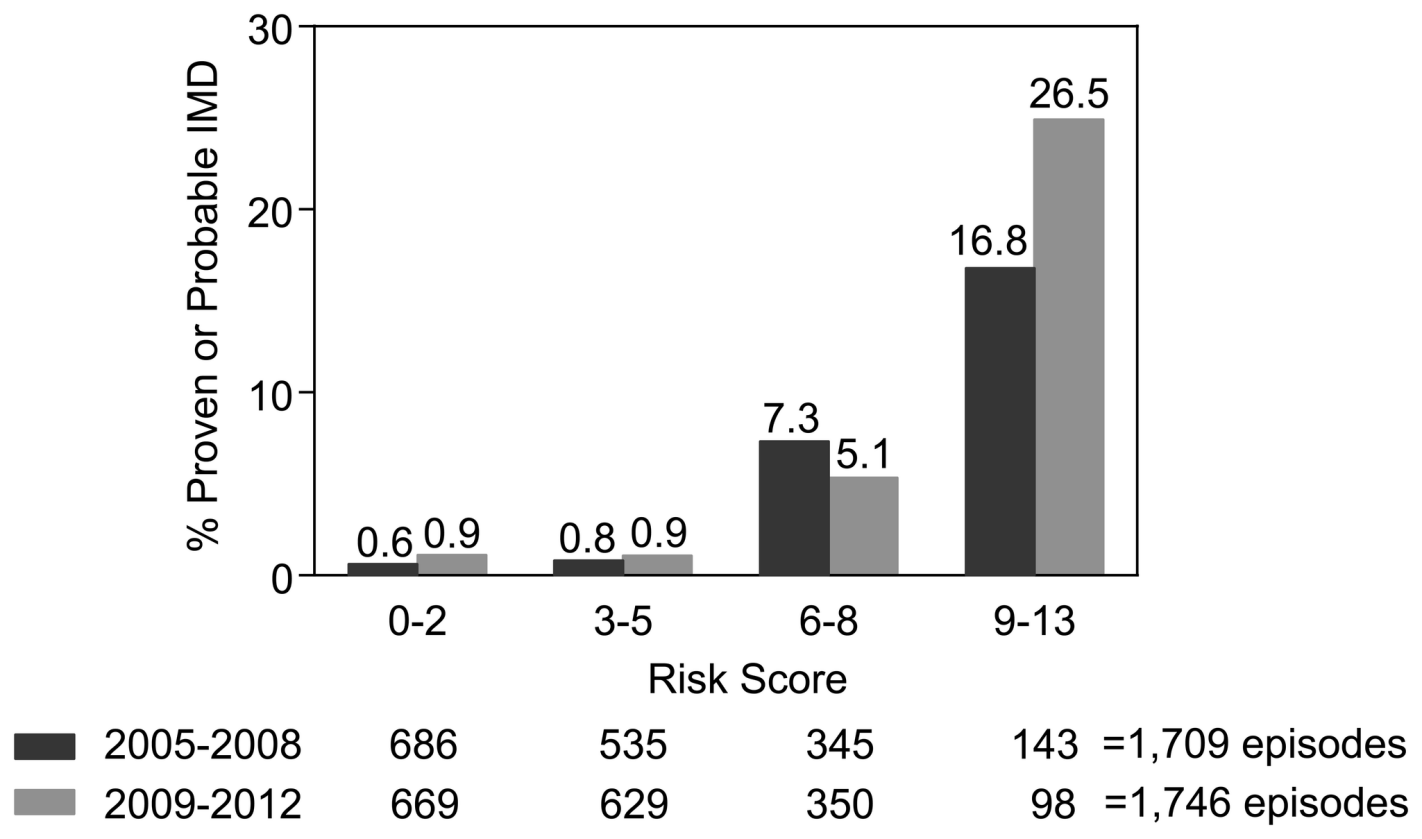
Distribution of risk scores versus the cumulative incidence of proven or probable invasive mold disease.

**Figure 2:**
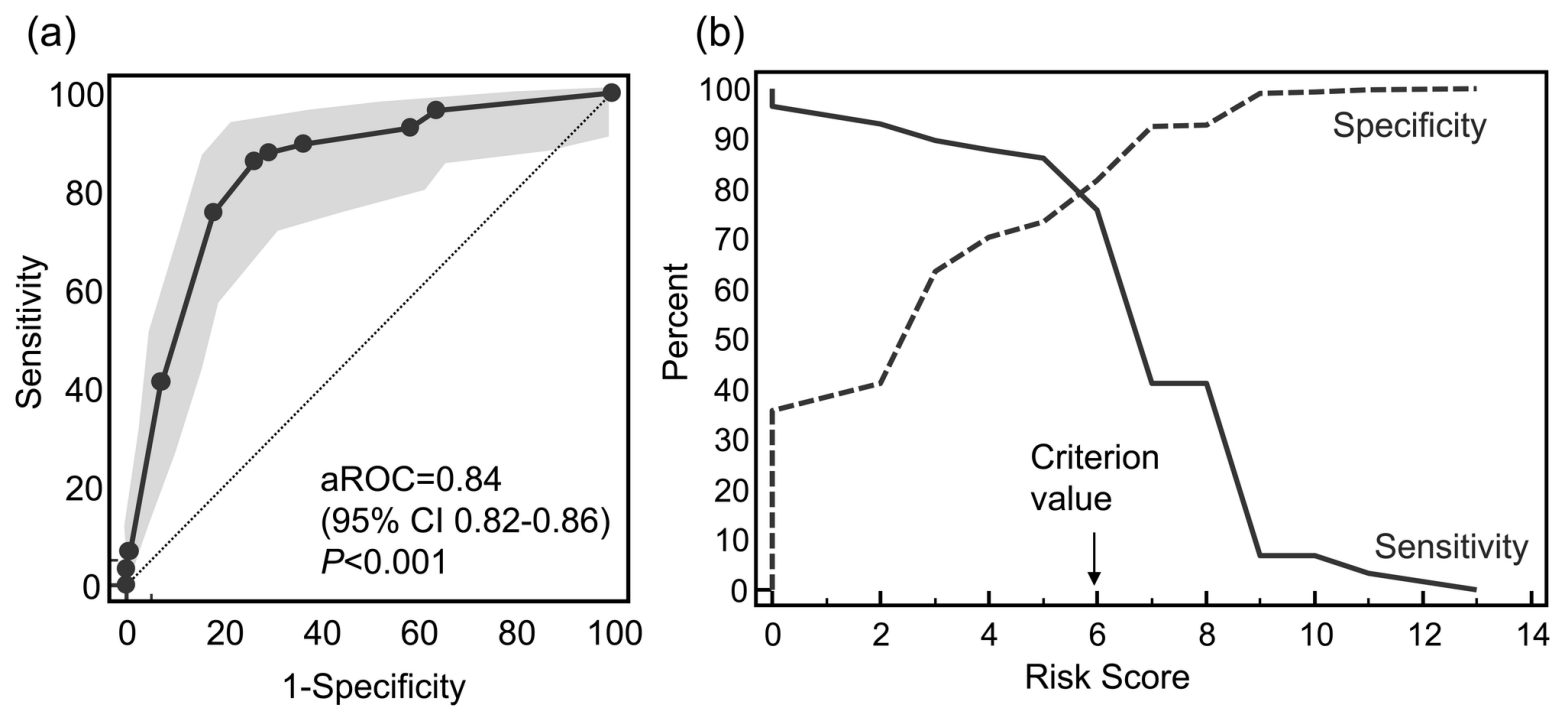
Analysis of risk score discrimination and optimal cut-off for invasive model disease risk. (a) Receiver-operator curve (ROC) analysis plot of the true positives plotted as a function of the false-positives (100-specificity) at different cutoffs of the risk score. Gray bands represent the 95% CI of the plot. The dotted line represents a reference line no discrimination for invasive mold disease (aROC=0.5). The *P* value is the probability that the aROC differs significantly from aROC=0.5; (b) Plot of sensitivity and specificity versus risk score. The highest sensitivity (true positive rate) and specificity (true negative rate) meet at a score just below 6, suggesting a criterion value of > 6.

Points assigned on the basis of the weighted odds ratios for these 4 independent variables resulted in a risk score from 0-13 for each patient (mean 3.3, 95% CI 3.1-3.4) (Table 4). Risk scores were well calibrated with observed rates of IMD (Figure 1). When risk scores and the rates of true-positive and false-positive IMD rates were analyzed by ROC curves (Figure 2), a score of less than 6 was found to be optimal cut-off for discriminating low-risk patients with an area under the ROC curve (aROC) of 0.84 (0.79-0.89), sensitivity 0.86 (0.77-0.95), specificity 0.74 (0.73-0.75), positive predictive value (PPV) 0.10 (0.07-0.13), and negative predictive value (NPV) of 0.99 (0.99-1).

The IMD risk score derived from multivariate analysis of the 2005-2008 cohort was calculated for each patient at the time of hospital admission during 2009-2012, and patients were monitored for the development of probable or proven IMD within 90 days or hospital discharge. The mean risk score in 2009-2012 (mean 3.1, 95% CI 3.0-3.3) did not differ significantly from patients analyzed from 2005-2008. Similar to the retrospective cohort, risk scores for patients studied during 2009-2012 were well calibrated with the incidence rate of IMD within 90 days of hospital admission (Figure 1). A score of less than 6 was also confirmed as the optimal cut-off for discriminating low-risk patients in the prospective study cohort, with an aROC of 0.84 (0.82-0.86), sensitivity of 0.80 (0.67-0.89), specificity 0.76 (0.74-0.78), PPV 0.10 (0.07-0.13) and NPV 0.99 (0.99-1.0). When the risk score performance was analyzed in different subgroups of hematological malignancy patients with varying IMD prevalence (1.5% to 10.6%) and rates of anti-mold prophylaxis use (7.2% to 57%), we found that a score of < 6 consistently identified a cohort of patients at low risk for IMD with NPVs ranging from 0.96-0.99 (Table 5).

**Table 5:** Predictive performance of the risk score in the 2009-2012 validation cohort.

**Group**	**Median risk score**	**Anti-mold prophylaxis during episode**	**IMD prevalence**	**aROC(95% CI)**	**Sensitivity(95% CI)**	**Specificity(95% CI)**	**Positive predictive value (95% CI)**	**Negative predictive value (95% CI)**
All patientsn=1,746	3	20%	3.2%	0.84 (0.82-0.86)	0.80 (0.67-0.89)	0.76 (0.74-0.78)	0.10 (0.07-0.13)	0.99 (0.99-1.0)
Acute myeloid leukemia(remission-induction), n=131^*a*^	7	57%	6.1%	0.64 (0.55-0.72)	0.88 (0.47-0.99)	0.24 (0.17-0.33)	0.07 (0.03-0.14)	0.97 (0.83-0.99)
Acute myeloid leukemia (consolidation/salvage), n=284^*b*^	4	46%	1.4%	0.80 (0.75-0.85)	0.75 (0.19-0.99)	0.71 (0.65-0.76)	0.04 (0.007-.10)	0.99 (0.97-1.0)
Lymphoma, n=390^b^	3	7.2%	1.5%	0.99 (0.97-1.0)	1.0 (0.54-1.0)	0.94 (0.91-0.96)	0.20 (0.08-0.39)	0.99 (0.99-1.0)
Allogeneic HSCT, n=227	5	13%	10.6%	0.72 (0.65-0.77)	0.88 (0.68-0.97)	0.33 (0.26-0.39)	0.13 (0.8-0.20)	0.96 (0.88-0.99)

a Only first admission for remission-induction chemotherapy was considered

b Excludes patients who received allogeneic or autologous HSCT

Note: Risk score performance for autologous HSCT is not shown in the table because only 1 case of IMD was documented in 344 admissions

### Impact of Posaconazole Prophylaxis

**Figure 3:**
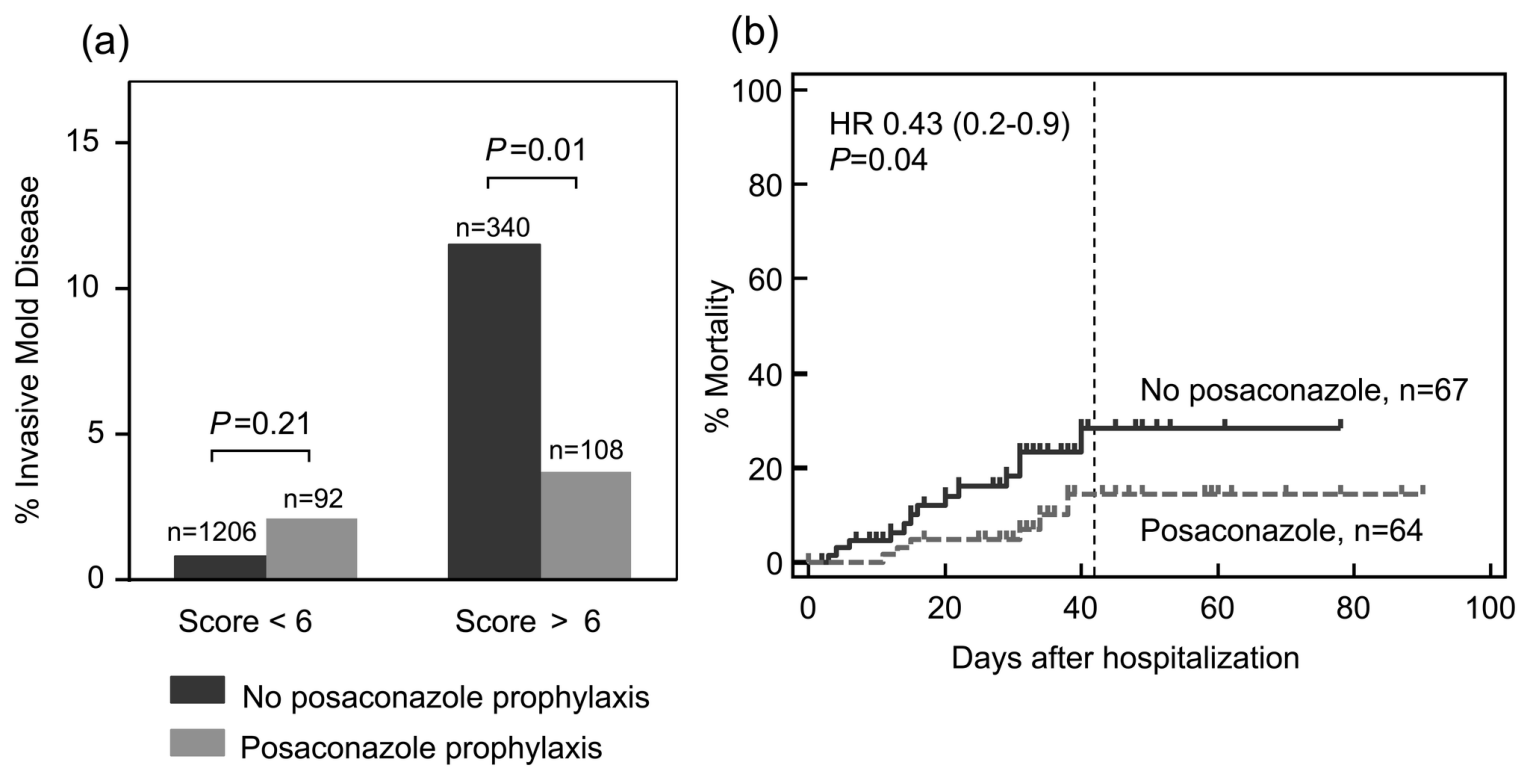
Impact of posaconazole prophylaxis on the incidence and mortality of invasive mold disease in the 2009-2012 validation cohort. (a) Cumulative incidence of invasive mold disease in patients with calculated risk scores <6 or > 6. *P* value determined by Chi-square test. (b) Kaplan-Meier analysis of crude mortality in patients with acute myelogenous leukemia or myelodysplastic syndrome undergoing remission-induction chemotherapy by status of posaconazole prophylaxis. Each patient is analyzed only once and was classified as alive or dead at the time of discharge (censored) or death by day +42 after admission. *P* value was determined by the Mantel-Cox (log-rank) test.

Posaconazole prophylaxis reduces the incidence of IMD in high-risk hematology patients and was associated with a mortality benefit in AML/MDS patients receiving remission- induction chemotherapy [13,14]. We examined rates of proven or probable IMD among patients who had received posaconazole with risk scores of < 6 versus > 6 (Figure 3a). Posaconazole prophylaxis was not associated with any discernable benefit in terms of reducing the incidence of IMD in patients with risk scores of < 6. However, among higher-risk patients with scores > 6, posaconazole prophylaxis was associated with a 7.8% risk reduction in IMD (*P*=0.01). We also found that among 131 individual patients with AML/MDS undergoing remission-induction chemotherapy during 2009-2012, patients who received posaconazole prophylaxis had a significantly lower risk of crude mortality within 6 weeks of hospitalization [15], versus patients who did not receive posaconazole (Figure 3b) (HR 0.43, 0.2-0.9, *P*=0.04). This mortality difference was evident despite identical median risk scores (7) in patients who received and did not receive posaconazole prophylaxis.

## Discussion

Physicians must weigh multiple factors when considering a patient’s risk for developing IMD [16,17]. Prognostic models or risk scores can complement this clinical assessment by providing an objective summation of multiple risk factors, thereby clarifying which patients should be targeted for more aggressive intervention [18]. To our knowledge, our single-institution study represents the first attempt to develop and validate an unconditional risk model for IMD in a heterogeneous population of patients with hematological malignancies. Our data demonstrate that an objective weighted risk score could reliably discriminate patients who had a very low probability of developing IMD within 90 days of hospitalization, and thus may be candidates for more conservative management with respect to higher-risk patients.

An ideal risk score for IMD in patients would have both a high negative predictive value (NPV) and high positive predictive value (PPV). Yet, development of such a risk score that could be applied for routine screening of a heterogeneous population of hematology patients is challenging, given the overall low prevalence of IMD and fluctuating risk factors for infection [8]. Alternatively, a risk score could be developed in a more homogenous high-risk population of with a higher prevalence of IMD (

> 5%), such as allogeneic HSCT or AML/MDS patients undergoing remission-induction chemotherapy. Risk scores targeting populations who have already been shown to have proven to benefit from antifungal prophylaxis or intensive monitoring, however, may have less clinical utility for routine patient care [18] or considered too restrictive by treating physicians [19].

A limitation of our study is that our risk score was devised from observational data in a single center, and could not control for “real life” confounding factors such as use of antifungal prophylaxis. Notably, the discriminative performance of the risk score in our institution was similar among various subgroups of hematology malignancy patients with varying risk for IMD and usage patterns of antifungal prophylaxis. However, the performance of our risk score will undoubtedly vary in other hospitals depending on the type of patients treated and the baseline incidence of IMD. Additionally, clinical risk factors for IMD such as graft versus host disease and corticosteroids, which were not retained in our final risk model, would likely be more important if the score was developed specifically in allogeneic HSCT patients. Therefore multicenter validation and center-specific adjustments would likely be required if the risk score was applied to the clinical management of IMD in other hospitals.

In conclusion, we found than an objective, weighted risk-score for IMD could reliably discriminate the large majority of patients with hematological malignancies who were at low-risk for developing IMD. The discriminative performance of the score was consistent across various hematology patient subtypes with varying underlying baseline risk for IMD and exposure to antifungal prophylaxis. The continued refinement and multicenter validation of IMD risk scores could complement the clinical assessment of patients with hematological malignancies, and possibly improve the targeted use of diagnostics and antifungals in this immunocompromised population.

## References

[1] Pagano L, Caira M, Candoni A, Offidani M, Martino B et al. (2010) Invasive aspergillosis in patients with acute myeloid leukemia: a SEIFEM-2008 registry study. Haematologica 95: 644-650. doi:10.3324/haematol.2009.012054. PubMed: 19850903. 19850903 10.3324/haematol.2009.012054 PMC2857195

[2] Upton A, Kirby KA, Carpenter P, Boeckh M, Marr KA (2007) Invasive aspergillosis following hematopoietic cell transplantation: outcomes and prognostic factors associated with mortality. Clin Infect Dis 44: 531-540. doi:10.1086/510592. PubMed: 17243056. 17243056 10.1086/510592

[3] Park BJ, Pappas PG, Wannemuehler KA, Alexander BD, Anaissie EJ et al. (2011) Invasive non-Aspergillus mold infections in transplant recipients, United States, 2001-2006. Emerg Infect Dis 17: 1855-1864. doi:10.3201/eid1710.110087. PubMed: 22000355. 22000355 10.3201/eid1710.110087 PMC3311117

[4] Pagano L, Caira M, Candoni A, Offidani M, Fianchi L et al. (2006) The epidemiology of fungal infections in patients with hematologic malignancies: the SEIFEM-2004 study. Haematologica 91: 1068-1075. PubMed: 16885047. 16885047

[5] de Pauw BE (2005) Between over- and undertreatment of invasive fungal disease. Clin Infect Dis 41: 1251-1253. doi:10.1086/496933. PubMed: 16206098. 16206098 10.1086/496933

[6] de Pauw BE, Viscoli C (2011) Managing invasive fungal infections: relying on clinical instincts or on a rational navigation system? J Antimicrob Chemother 66 Suppl 1: i55-i58. doi:10.1093/jac/dkr125. PubMed: 21177405. 21177405 10.1093/jac/dkq442

[7] Freifeld AG, Bow EJ, Sepkowitz KA, Boeckh MJ, Ito JI et al. (2011) Clinical practice guideline for the use of antimicrobial agents in neutropenic patients with cancer: 2010 update by the Infectious Diseases Society of America. Clin Infect Dis 52: e56-e93. doi:10.1093/cid/cir073. PubMed: 21258094. 21258094 10.1093/cid/cir073

[8] Herbrecht R, Bories P, Moulin JC, Ledoux MP, Letscher-Bru V (2012) Risk stratification for invasive aspergillosis in immunocompromised patients. Ann N Y Acad Sci 1272: 23-30. doi:10.1111/j.1749-6632.2012.06829.x. PubMed: 23231711. 23231711 10.1111/j.1749-6632.2012.06829.x

[9] van der Velden WJ, Blijlevens NM, Donnelly JP (2011) Genetic variants and the risk for invasive mould disease in immunocompromised hematology patients. Curr Opin Infect Dis 24: 554-563. doi:10.1097/QCO.0b013e32834ab1f4. PubMed: 21926619. 21926619 10.1097/QCO.0b013e32834ab1f4

[10] De Pauw B, Walsh TJ, Donnelly JP, Stevens DA, Edwards JE et al. (2008) Revised definitions of invasive fungal disease from the European Organization for Research and Treatment of Cancer/Invasive Fungal Infections Cooperative Group and the National Institute of Allergy and Infectious Diseases Mycoses Study Group (EORTC/MSG) Consensus Group. Clin Infect Dis 46: 1813-1821. doi:10.1086/588660. PubMed: 18462102. 18462102 10.1086/588660 PMC2671227

[11] Cornely OA, Maertens J, Bresnik M, Ebrahimi R, Ullmann AJ et al. (2007) Liposomal amphotericin B as initial therapy for invasive mold infection: a randomized trial comparing a high-loading dose regimen with standard dosing (AmBiLoad trial). Clin Infect Dis 44: 1289-1297. doi:10.1086/514341. PubMed: 17443465. 17443465 10.1086/514341

[12] Rogers TR, Slavin MA, Donnelly JP (2011) Antifungal prophylaxis during treatment for haematological malignancies: are we there yet? Br J Haematol 153: 681-697. doi:10.1111/j.1365-2141.2011.08650.x. PubMed: 21504422. 21504422 10.1111/j.1365-2141.2011.08650.x

[13] Cornely OA, Maertens J, Winston DJ, Perfect J, Ullmann AJ et al. (2007) Posaconazole vs. fluconazole or itraconazole prophylaxis in patients with neutropenia. N Engl J Med 356: 348-359. doi:10.1056/NEJMoa061094. PubMed: 17251531. 17251531 10.1056/NEJMoa061094

[14] Ullmann AJ, Lipton JH, Vesole DH, Chandrasekar P, Langston A et al. (2007) Posaconazole or fluconazole for prophylaxis in severe graft-versus-host disease. N Engl J Med 356: 335-347. doi:10.1056/NEJMoa061098. PubMed: 17251530. 17251530 10.1056/NEJMoa061098

[15] Wingard JR, Ribaud P, Schlamm HT, Herbrecht R (2008) Changes in causes of death over time after treatment for invasive aspergillosis. Cancer 112: 2309-2312. doi:10.1002/cncr.23441. PubMed: 18338758. 18338758 10.1002/cncr.23441

[16] Moons KG, Royston P, Vergouwe Y, Grobbee DE, Altman DG (2009) Prognosis and prognostic research: what, why, and how? BMJ 338: b375. doi:10.1136/bmj.b375. PubMed: 19237405. 19237405 10.1136/bmj.b375

[17] Royston P, Moons KG, Altman DG, Vergouwe Y (2009) Prognosis and prognostic research: Developing a prognostic model. BMJ 338: b604. doi:10.1136/bmj.b604. PubMed: 19336487. 19336487 10.1136/bmj.b604

[18] Moons KG, Altman DG, Vergouwe Y, Royston P (2009) Prognosis and prognostic research: application and impact of prognostic models in clinical practice. BMJ 338: b606. doi:10.1136/bmj.b606. PubMed: 19502216. 19502216 10.1136/bmj.b606

[19] Ostrosky-Zeichner L, Pappas PG, Shoham S, Reboli A, Barron MA et al. (2011) Improvement of a clinical prediction rule for clinical trials on prophylaxis for invasive candidiasis in the intensive care unit. Mycoses 54: 46-51. doi:10.1111/j.1439-0507.2009.01756.x. PubMed: 19627509. 19627509 10.1111/j.1439-0507.2009.01756.x

[20] O’Brien SN, Blijlevens NM, Mahfouz TH, Anaissie EJ (2003) Infections in patients with hematological cancer: recent developments. Hematology Am Soc Hematol Educ Program 2003: 438-472. 14633794 10.1182/asheducation-2003.1.438

[21] Robin M, Porcher R, De Castro Araujo R, de Latour RP, Devergie A et al. (2007) Risk factors for late infections after allogeneic hematopoietic stem cell transplantation from a matched related donor. Biol Blood Marrow Transplant 13: 1304-1312. doi:10.1016/j.bbmt.2007.07.007. PubMed: 17950917. 17950917 10.1016/j.bbmt.2007.07.007

[22] Verweij PE, Kerremans JJ, Voss A, Meis JF (2000) Fungal contamination of tobacco and marijuana. JAMA 284: 2875. doi:10.1001/jama.284.22.2869. PubMed: 11147983. 11147983 10.1001/jama.284.22.2875

[23] Grigg A, Slavin M (2008) Minimizing the risk of recurrent or progressive invasive mold infections during stem cell transplantation or further intensive chemotherapy. Transpl Infect Dis 10: 3-12. doi:10.1111/j.1399-3062.2007.00259.x. PubMed: 17605732. 17605732 10.1111/j.1399-3062.2007.00259.x

[24] Post MJ, Lass-Floerl C, Gastl G, Nachbaur D (2007) Invasive fungal infections in allogeneic and autologous stem cell transplant recipients: a single-center study of 166 transplanted patients. Transpl Infect Dis 9: 189-195. doi:10.1111/j.1399-3062.2007.00219.x. PubMed: 17511828. 17511828 10.1111/j.1399-3062.2007.00219.x

[25] Marr KA, Carter RA, Boeckh M, Martin P, Corey L (2002) Invasive aspergillosis in allogeneic stem cell transplant recipients: changes in epidemiology and risk factors. Blood 100: 4358-4366. doi:10.1182/blood-2002-05-1496. PubMed: 12393425. 12393425 10.1182/blood-2002-05-1496

[26] El-Cheikh J, Castagna L, Wang L, Esterni B, Faucher C et al. (2010) Impact of prior invasive aspergillosis on outcome in patients receiving reduced-intensity conditioning allogeneic hematopoietic stem cell transplant. Leuk Lymphoma 51: 1705-1710. PubMed: 20629522. 20629522 10.3109/10428194.2010.500433

[27] Vazquez JA, Sobel JD (1995) Fungal infections in diabetes. Infect Dis Clin North Am 9: 97-116. PubMed: 7769222. 7769222

[28] Soubani AO, Qureshi MA (2002) Invasive pulmonary aspergillosis following bone marrow transplantation: risk factors and diagnostic aspect. Haematologia (Budap) 32: 427-437. PubMed: 12803117. 12803117

[29] Garcia-Vidal C, Upton A, Kirby KA, Marr KA (2008) Epidemiology of invasive mold infections in allogeneic stem cell transplant recipients: biological risk factors for infection according to time after transplantation. Clin Infect Dis 47: 1041-1050. doi:10.1086/591969. PubMed: 18781877. 18781877 10.1086/591969 PMC2668264

[30] Lionakis MS, Kontoyiannis DP (2003) Glucocorticoids and invasive fungal infections. Lancet 362: 1828-1838. doi:10.1016/S0140-6736(03)14904-5. PubMed: 14654323. 14654323 10.1016/S0140-6736(03)14904-5

[31] Garnacho-Montero J, Amaya-Villar R, Ortiz-Leyba C, León C, Alvarez-Lerma F et al. (2005) Isolation of Aspergillus spp. from the respiratory tract in critically ill patients: risk factors, clinical presentation and outcome. Crit Care 9: R191-R199. doi:10.1186/cc3254. PubMed: 15987390. 15987390 10.1186/cc3488 PMC1175876

[32] Gavalda J, Len O, San Juan R, Aguado JM, Fortun J et al. (2005) Risk factors for invasive aspergillosis in solid-organ transplant recipients: a case-control study. Clin Infect Dis 41: 52-59. doi:10.1086/430602. PubMed: 15937763. 15937763 10.1086/430602

[33] Mühlemann K, Wenger C, Zenhäusern R, Täuber MG (2005) Risk factors for invasive aspergillosis in neutropenic patients with hematologic malignancies. Leukemia 19: 545-550. PubMed: 15729382. 15729382 10.1038/sj.leu.2403674

[34] Allam MF, Del Castillo AS, Diaz-Molina C, Navajas RF (2002) Invasive pulmonary aspergillosis: identification of risk factors. Scand J Infect Dis 34: 819-822. 12578151 10.1080/0036554021000026954

[35] Rondinelli PI, Ribeiro Kde C, de Camargo B (2006) A proposed score for predicting severe infection complications in children with chemotherapy-induced febrile neutropenia. J Pediatr Hematol/Oncol 28: 665-670. doi:10.1097/01.mph.0000212996.94929.0b. 10.1097/01.mph.0000212996.94929.0b 17023827

[36] Martino R, Parody R, Fukuda T, Maertens J, Theunissen K et al. (2006) Impact of the intensity of the pretransplantation conditioning regimen in patients with prior invasive aspergillosis undergoing allogeneic hematopoietic stem cell transplantation: A retrospective survey of the Infectious Diseases Working Party of the European Group for Blood and Marrow Transplantation. Blood 108: 2928-2936. doi:10.1182/blood-2006-03-008706. PubMed: 16720833. 16720833 10.1182/blood-2006-03-008706 PMC1895522

[37] Uys A, Rapoport BL, Anderson R (2004) Febrile neutropenia: a prospective study to validate the Multinational Association of Supportive Care of Cancer (MASCC) risk-index score. Support Care Cancer 12: 555-560. PubMed: 15197637. 15197637 10.1007/s00520-004-0614-5

[38] Gerson SL, Talbot GH, Hurwitz S, Strom BL, Lusk EJ et al. (1984) Prolonged granulocytopenia: the major risk factor for invasive pulmonary aspergillosis in patients with acute leukemia. Ann Intern Med 100: 345-351. doi:10.7326/0003-4819-100-3-345. PubMed: 6696356. 6696356 10.7326/0003-4819-100-3-345

[39] Thursky K, Byrnes G, Grigg A, Szer J, Slavin M (2004) Risk factors for post-engraftment invasive aspergillosis in allogeneic stem cell transplantation. Bone Marrow Transplant 34: 115-121. doi:10.1038/sj.bmt.1704543. PubMed: 15156166. 15156166 10.1038/sj.bmt.1704543

[40] Fukuda T, Boeckh M, Carter RA, Sandmaier BM, Maris MB et al. (2003) Risks and outcomes of invasive fungal infections in recipients of allogeneic hematopoietic stem cell transplants after nonmyeloablative conditioning. Blood 102: 827-833. doi:10.1182/blood-2003-02-0456. PubMed: 12689933. 12689933 10.1182/blood-2003-02-0456

[41] Glucksberg H, Storb R, Fefer A, Buckner CD, Neiman PE et al. (1974) Clinical manifestations of graft-versus-host disease in human recipients of marrow from HL-A-matched sibling donors. Transplantation 18: 295-304. doi:10.1097/00007890-197410000-00001. PubMed: 4153799. 4153799 10.1097/00007890-197410000-00001

[42] Labbé AC, Su SH, Laverdière M, Pépin J, Patiño C et al. (2007) High incidence of invasive aspergillosis associated with intestinal graft-versus-host disease following nonmyeloablative transplantation. Biol Blood Marrow Transplant 13: 1192-1200. doi:10.1016/j.bbmt.2007.06.013. PubMed: 17889356. 17889356 10.1016/j.bbmt.2007.06.013

[43] Shulman HM, Sullivan KM, Weiden PL, McDonald GB, Striker GE et al. (1980) Chronic graft-versus-host syndrome in man. A long-term clinicopathologic study of 20 Seattle patients. Am J Med 69: 204-217. doi:10.1016/0002-9343(80)90380-0. PubMed: 6996481. 6996481 10.1016/0002-9343(80)90380-0

[44] Parulekar W, Mackenzie R, Bjarnason G, Jordan RC (1998) Scoring oral mucositis. Oral Oncol 34: 63-71. doi:10.1016/S1368-8375(97)00065-1. PubMed: 9659522. 9659522 10.1016/s1368-8375(97)00065-1

[45] Grow WB, Moreb JS, Roque D, Manion K, Leather H et al. (2002) Late onset of invasive aspergillus infection in bone marrow transplant patients at a university hospital. Bone Marrow Transplant 29: 15-19. doi:10.1038/sj.bmt.1703332. PubMed: 11840139. 11840139 10.1038/sj.bmt.1703332

[46] Mahieu LM, De Dooy JJ, Van Laer FA, Jansens H, Ieven MM (2000) A prospective study on factors influencing Aspergillus spore load in the air during renovation works in a neonatal intensive care unit. J Hosp Infect 45: 191-197. doi:10.1053/jhin.2000.0773. PubMed: 10896797. 10896797 10.1053/jhin.2000.0773

[47] Cornet M, Levy V, Fleury L, Lortholary J, Barquins S et al. (1999) Efficacy of prevention by high-efficiency particulate air filtration or laminar airflow against *Aspergillus* airborne contamination during hospital renovation. Infect Control Hosp Epidemiol 20: 508-513. doi:10.1086/501661. PubMed: 10432165. 10432165 10.1086/501661

[48] Berthelot P, Loulergue P, Raberin H, Turco M, Mounier C et al. (2006) Efficacy of environmental measures to decrease the risk of hospital-acquired aspergillosis in patients hospitalised in haematology wards. Clin Microbiol Infect 12: 738-744. doi:10.1111/j.1469-0691.2006.01499.x. PubMed: 16842568. 16842568 10.1111/j.1469-0691.2006.01499.x

[49] Thio CL, Smith D, Merz WG, Streifel AJ, Bova G et al. (2000) Refinements of environmental assessment during an outbreak investigation of invasive aspergillosis in a leukemia and bone marrow transplant unit. Infect Control Hosp Epidemiol 21: 18-23. doi:10.1086/501691. PubMed: 10656349. 10656349 10.1086/501691

